# Correction: Impact of the COVID-19 pandemic on exercise habits and overweight status in Japan: a nation-wide panel survey

**DOI:** 10.1371/journal.pgph.0002653

**Published:** 2023-11-15

**Authors:** Sae Ochi, Mirai So, Sora Hashimoto, Yuki Hashimoto, Yoichi Sekizawa

The second author’s name is incorrect. The correct name is: Mirai So. The publisher apologizes for the error. The correct citation is: Ochi S, So M, Hashimoto S, Hashimoto Y, Sekizawa Y (2023) Impact of the COVID-19 pandemic on exercise habits and overweight status in Japan: A nation-wide panel survey. PLOS Glob Public Health 3(7): e0001732. https://doi.org/10.1371/journal.pgph.0001732
