# Supplementary material for: Impact of the COVID-19 pandemic on exercise habits and overweight status in Japan: A nation-wide panel survey
Source: PLOS Glob Public Health. 2023 Jul 19;3(7):e0001732. doi: 10.1371/journal.pgph.0001732 (PMC10355423; doi:10.1371/journal.pgph.0001732)
Supplement: S1 Table — (DOCX) [file pgph.0001732.s001.docx]

**S1 Table. Background of the participants who dropped out during the surveillance period.** Difference between dropout and non-dropout groups are calculated by chi-squared test

|  | | Missing data　(N=5,177) | | Complete data　(N=11,465) | | p |
| --- | --- | --- | --- | --- | --- | --- |
|  | | N | % | N | % |  |
| Female | | 3,134 | 61.2 | 5,486 | 47.8 | <0.01 |
| Age group | <=30 | 1,872 | 36.6 | 941 | 8.2 | <0.01 |
|  | 30-39 | 882 | 17.2 | 1,171 | 10.2 |  |
|  | 40-49 | 718 | 14.0 | 2,538 | 22.1 |  |
|  | 50-59 | 616 | 12.0 | 2,700 | 23.5 |  |
|  | 60-69 | 774 | 15.1 | 2,806 | 24.5 |  |
|  | 70-74 | 315 | 6.2 | 1,309 | 11.4 |  |
| Income (1,000yen/year)* | <300 | 1,427 | 27.9 | 2,865 | 25.0 | <0.01 |
|  | 300-500 | 1,512 | 29.5 | 3,077 | 26.8 |  |
|  | 500-700 | 924 | 18.1 | 2,255 | 19.7 |  |
|  | 700-1000 | 835 | 16.3 | 1,960 | 17.1 |  |
|  | >1000 | 479 | 9.4 | 1,308 | 11.4 | <0.01 |
| Marital status | Married | 2,609 | 51.0 | 7,296 | 63.6 | <0.01 |
|  | Divorced | 245 | 4.8 | 704 | 6.1 |  |
|  | Widow/widower | 83 | 1.6 | 283 | 2.5 |  |
|  | Never married | 2,240 | 43.8 | 3,182 | 27.8 |  |
| Pre-existing condition | Obesity | 758 | 14.8 | 1,828 | 15.9 | 0.03 |
|  | High blood pressure | 536 | 10.5 | 1,993 | 17.4 | <0.01 |
|  | Lipid abnormalities | 311 | 6.1 | 1,152 | 10.0 | <0.01 |
|  | Diabetes | 172 | 3.4 | 624 | 5.4 | <0.01 |
|  | Heart disease | 88 | 1.7 | 290 | 2.5 | <0.01 |
|  | Lung or respiratory disease | 122 | 2.4 | 254 | 2.2 | 0.57 |
|  | Renal disease | 28 | 0.5 | 92 | 0.8 | <0.01 |
|  | Cancer | 58 | 1.1 | 184 | 1.6 | 0.02 |
|  | Depression | 1,133 | 22.1 | 1,644 | 14.3 | <0.01 |
|  | Other condition* | 74 | 1.4 | 161 | 1.4 | <0.01 |

*1yen≒110-130$

** Disease due to which the participant are prohibited by a doctor from exercising, or disease or injury due to which you have major difficulties in walking (e.g., rheumatoid arthritis and bone fracture)
