# Supplementary material for: Impact of the COVID-19 pandemic on exercise habits and overweight status in Japan: A nation-wide panel survey
Source: PLOS Glob Public Health. 2023 Jul 19;3(7):e0001732. doi: 10.1371/journal.pgph.0001732 (PMC10355423; doi:10.1371/journal.pgph.0001732)
Supplement: S2 Table — (DOCX) [file pgph.0001732.s002.docx]

**S2 Table. Impact of the factors on prolonged overweight in the early phase of the pandemic.** Controlled for pre-existing condition.

|  | | Male | | | Female | | |
| --- | --- | --- | --- | --- | --- | --- | --- |
|  |  | OR | 95%CI | *p* | OR | 95%CI | *p* |
| Past diagnosis of COVID-19 | | 3.01 | 1.27, 7.13 | 0.01† | NC | | |
| Age group  (yr) | <=30 | 1 (Reference) | | | 1 (Reference) | | |
|  | 31-40 | 1.63 | 0.75, 3.54 | 0.22 | 0.53 | 0.20, 1.35 | 0.18 |
|  | 41-50 | 2.31 | 1.14, 4.64 | 0.02† | 0.58 | 0.26, 1.28 | 0.18 |
|  | 51-60 | 2.12 | 1.04, 4.31 | 0.04† | 0.59 | 0.27, 1.30 | 0.19 |
|  | 61-70 | 1.99 | 0.96, 4.16 | 0.07 | 0.67 | 0.30, 1.47 | 0.32 |
|  | 71-80 | 1.27 | 0.57, 2.84 | 0.56 | 0.95 | 0.40, 2.24 | 0.91 |
| Income (yen/year)* 1yen≒110-130$ | <300 | 1 (Reference) | | | 1 (Reference) | | |
|  | 300-500 | 1.11 | 0.77, 1.59 | 0.58 | 1.00 | 0.59, 1.70 | 0.99 |
|  | 500-700 | 1.07 | 0.71, 1.59 | 0.76 | 1.19 | 0.66, 2.14 | 0.56 |
|  | 700-1000 | 1.17 | 0.78, 1.75 | 0.46 | 0.89 | 0.45, 1.77 | 0.74 |
|  | >1000 | 1.19 | 0.76, 1.84 | 0.44 | 1.29 | 0.63, 2.67 | 0.49 |
| Married | | 1.17 | 0.87, 1.59 | 0.30 | 1.29 | 0.81, 2.05 | 0.28 |
| Income change | No change | 1 (Reference) | | | 1 (Reference) | | |
|  | Decrease | 0.73 | 0.54, 0.97 | 0.03† | 1.75 | 1.18, 2.61 | 0.01† |
|  | Increase | 0.99 | 0.56, 1.75 | 0.98 | 0.34 | 0.05, 2.53 | 0.30 |
| Lifestyle | Avoid poorly ventilated places | 1.41 | 0.93, 2.13 | 0.10 | 1.42 | 0.56, 3.56 | 0.46 |
|  | Avoid places where many people gather | 0.79 | 0.52, 1.20 | 0.27 | 0.56 | 0.26, 1.20 | 0.14 |
|  | Avoid talking at close distances | 0.85 | 0.60, 1.22 | 0.38 | 1.29 | 0.67, 2.48 | 0.44 |
|  | Wear a mask | 1.90 | 0.92, 3.93 | 0.08 | NC | | |
|  | Wash hands | 0.56 | 0.32, 0.97 | 0.04† | 0.98 | 0.13, 7.63 | 0.98 |
|  | Change clothes frequently | 0.93 | 0.65, 1.34 | 0.71 | 1.60 | 0.99, 2.59 | 0.06 |
|  | Gargle | 0.99 | 0.75, 1.30 | 0.94 | 1.14 | 0.71, 1.84 | 0.59 |
|  | Disinfect belongings | 1.15 | 0.83, 1.60 | 0.40 | 0.65 | 0.40, 1.05 | 0.08 |
|  | Keep distance from others | 0.79 | 0.56, 1.13 | 0.21 | 1.01 | 0.51, 2.00 | 0.97 |
|  | Refrain from seeing a doctor | 0.86 | 0.65, 1.13 | 0.28 | 0.75 | 0.49, 1.16 | 0.20 |
|  | Refrain from going out | 1.16 | 0.89, 1.53 | 0.28 | 1.51 | 0.95, 2.41 | 0.08 |
|  | Exercise regularly | 1.15 | 0.89, 1.49 | 0.29 | 1.11 | 0.74, 1.66 | 0.62 |
| Subjective Health | Very good | 1 (Reference) | | | 1 (Reference) | | |
|  | Good | 0.84 | 0.51, 1.37 | 0.48 | 0.66 | 0.29, 1.53 | 0.34 |
|  | Relatively good | 0.85 | 0.51, 1.41 | 0.53 | 1.06 | 0.46, 2.45 | 0.89 |
|  | Relatively bad | 0.89 | 0.50, 1.60 | 0.71 | 1.12 | 0.42, 2.97 | 0.82 |
|  | Bad | 0.76 | 0.34, 1.70 | 0.50 | 1.13 | 0.25, 5.06 | 0.87 |
|  | Very bad | 1.11 | 0.28, 4.40 | 0.88 | 6.36 | 0.95, 42.51 | 0.06 |
| PHQ-9 | | 1.01 | 0.97, 1.06 | 0.61 | 0.99 | 0.91, 1.07 | 0.74 |
| GAD-7 | | 1.00 | 0.95, 1.06 | 0.96 | 0.96 | 0.88, 1.05 | 0.43 |

*1yen≒110-130USD, † *p*<0.05

**Figure S1. Plot of the body mass index of each participant in October 2020 (horizontal) and October 2021 (vertical).**
