# Supplementary figures and images for: Impact of the COVID-19 pandemic on exercise habits and overweight status in Japan: A nation-wide panel survey
Source: PLOS Glob Public Health. 2023 Jul 19;3(7):e0001732. doi: 10.1371/journal.pgph.0001732 (PMC10355423; doi:10.1371/journal.pgph.0001732)

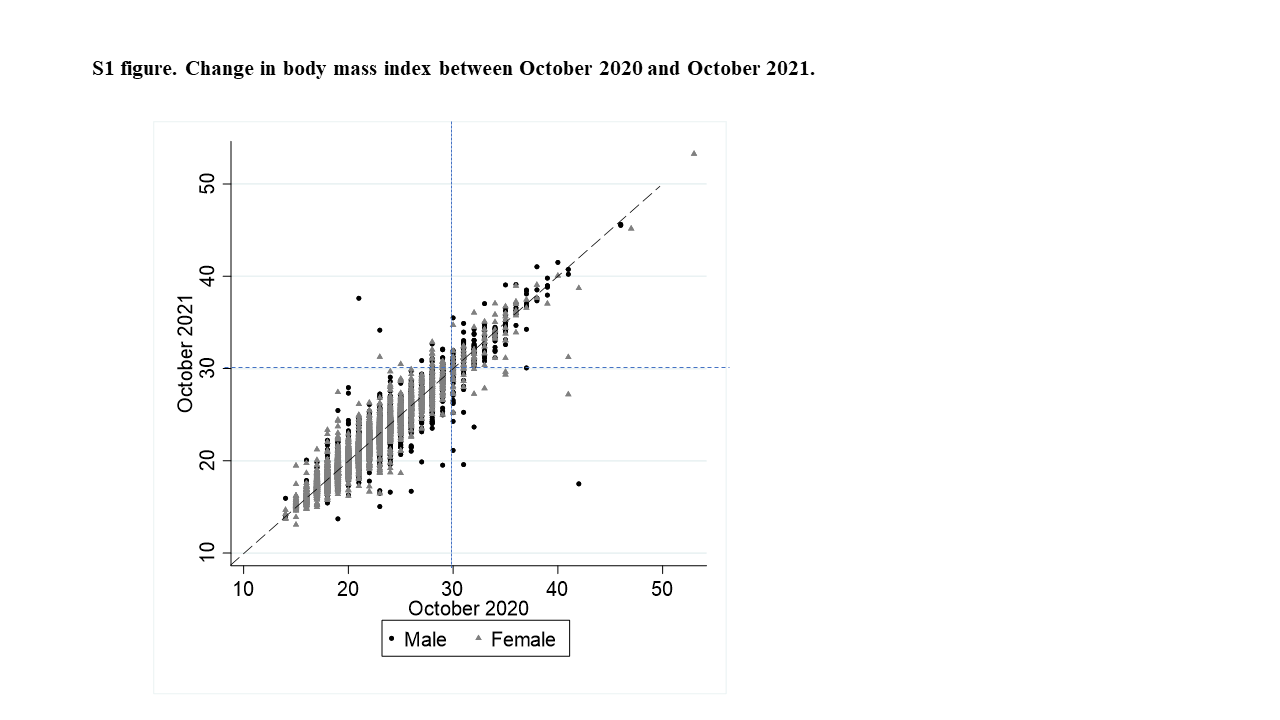

Supplement: S1 Fig — (TIF) [file pgph.0001732.s003.TIF]
