# Supplementary material for: Impact of the COVID-19 pandemic on exercise habits and overweight status in Japan: A nation-wide panel survey
Source: PLOS Glob Public Health. 2023 Jul 19;3(7):e0001732. doi: 10.1371/journal.pgph.0001732 (PMC10355423; doi:10.1371/journal.pgph.0001732)
Supplement: Attachment — Submitted filename: ResponseToReviewersR1.docx [file pgph.0001732.s004.docx]

Response to the editor and the reviewer

To the Editor:

1. We suggest you thoroughly copyedit your manuscript for language usage, spelling, and grammar. If you do not know anyone who can help you do this, you may wish to consider employing a professional scientific editing service.

Response: We have had our manuscript copyedited by a native speaker again. Please see the attached proof of the proofread.

2. Please send a completed 'Competing Interests' statement, including any COIs declared by your co-authors. If you have no competing interests to declare, please state "The authors have declared that no competing interests exist". Otherwise please declare all competing interests beginning with the statement "I have read the journal's policy and the authors of this manuscript have the following competing interests:"

Response: We have reconfirmed all financial and non-financial competing interests for all the coauthors.

3. Please provide a/amend your detailed Financial Disclosure statement. This is published with the article. It must therefore be completed in full sentences and contain the exact wording you wish to be published.

a. Please clarify all sources of funding (financial or material support) for your study. List the grants (with grant number) or organizations (with url) that supported your study, including funding received from your institution.

b. State the initials, alongside each funding source, of each author to receive each grant.

c. State what role the funders took in the study. If the funders had no role in your study, please state: “The funders had no role in study design, data collection and analysis, decision to publish, or preparation of the manuscript.”

Response: We have reconfirmed the funding resource for all the coauthors.

4. Please provide separate figure files in .tif or .eps format.

Response: We have uploaded figure files including supplementary figures in tif format.

5. We noticed that you used “data not shown”/"unpublished data" in the manuscript. We do not allow these references, as the PLOS data access policy requires that all data be either published with the manuscript or made available in a publicly accessible database. Please amend the supplementary material to include the referenced data or remove the references.

Response:　We have amended the description so that it include the results of our calculations.

“Average days of exercise per week in those who were infected and those who were not were 2.30 days and 2.00 days in the early phase (p=0.16 by t-test) and 2.43 days and 2.13 days in the late phase(p=0.13)” (Page 23, Line 6-8)

6. Since your data is not available for proprietary reasons, please explain via email why the data is not available.

Response: We have asked the RIETI about data availability, and they answered that the data is available for request. So we have added the following description.

“The data used in the present study belong to the RIETI and can be obtained from the institute upon reasonable request.”

7. We have noticed that you have uploaded Supporting Information files, but you have not included a list of legends. Please add a full list of legends for your Supporting Information files after the references list.

Response: we have added the legends of the supporting information files.

“Supporting information

S1 Table. Background of the participants who dropped out during the surveillance period.

S2 Table. Impact of the factors on prolonged overweight in the early phase of the pandemic.

S1 Figure. Plot of the body mass index of each participant in October 2020 (horizontal) and October 2021 (vertical).” (Page 34, Line 12-16)

Reviewers' comments:

Reviewer #1: Interesting and important topic
- Not clear how the COVID-19 pandemic falls under the definition of CBRNE. Perhaps there is no need to put it under this category as it becomes vague and inaccurate.

Response: We agree our explanation about the relationship between pandemic and CBRNE disaster was insufficient. We have added the following sentence in Introduction.

“Another type of CBRNE disaster, biological disaster, is a disaster caused by the rapid spread of disease caused by microorganisms. As fear of invisible microorganisms can cause social panic, the situation similar to nuclear disaster may happen.” （Page 3, Line 16-18）

- make it more clear regarding correlation and not causation (use correlation instead of may cause), and add more on that in the limitations section.

Response: Thank you for this important advice. We have changed several words of causality into those of association. We have also added further sentence in the Limitation.

“Finally, there are many potential confounders that were not asked in the questionnaire. For example, decrease in exercise day does not always mean decline in activity. For example, before the pandemic the average commuting hours of Japanese businesspeople was about 50 minutes, which could be substitute for exercise time [37]. Therefore, it is possible that increase in working at home may have decreased overall activities even when the exercise day increased. To elucidate more detailed causal relationships, further data collection including physical performance tests is required. However, despite these limitations, our research provided sufficient generalizability compared to other studies because of the broadness of the participants’ background.” (Page 26, Line 8-15)

-Add what further studies are recommended to investigate this correlation/causation further - in the conclusion section.

Response: We have added the following sentence in conclusion as well as discussion.

“To clarify the causal relationships, further research is required such as long-term follow-up of the infected people.” (Page 24, Line 1-2)

“Therefore, it is possible that increase in working at home may have decreased overall activities even when the exercise day increased. To elucidate more detailed causal relationships, further research such as prospective study of physical performance tests is required.” (Page 26, Line 11-14)

“Further research is needed to clarify the detailed factors that may affect exercise habits and overweight status, such as eating habits.” (Page 27, Line 6-8)

-In discussion : add more on role of possible confounding factors that are missed in this study (e.g. eating habits).

Response: We have added several discussions in the manuscript.

“Another possible reason is that female whose income decreased were more likely to become depressive. Further research is required to elucidate the reasons why exercise times in females were not a trade-off for a reduction in income.” (Page 15, Line 3-8)

“Another scenario is that the development of overweight status has led to increase in the risk of symptomatic SARS-CoV-2 infection. As overweight status and obesity is a risk factor of developing severer symptoms, people in overweight status may have been at higher risks of being diagnosed as SARS-CoV-2 infection. To clarify the causal relationships, further research is required such as long-term follow-up of the infected people.” (Page 23, Line 17- Page 24, Line 2)

“Another possible reason is change in eating habit. If people try to go out as seldom as possible, they may buy more preserved food and less fresh fruits and vegetables, which may affect body weight. However, older age confounds with a variety of socio-economic and mental status such as living on pensions and dementia and further surveys on the impact of such factors on health status are required.” (Page 24, Line 12-17)

“In addition, several important questions that may affect body weight, such as eating habits, are not included in the questionnaire mainly due to lack of finance.” (Page 25, Line 14-15)
