# Supplementary material for: Impact of the COVID-19 pandemic on exercise habits and overweight status in Japan: A nation-wide panel survey
Source: PLOS Glob Public Health. 2023 Jul 19;3(7):e0001732. doi: 10.1371/journal.pgph.0001732 (PMC10355423; doi:10.1371/journal.pgph.0001732)
Supplement: Attachment — Submitted filename: RebuttalLetterR2.docx [file pgph.0001732.s005.docx]

Dear Editor and Reviewers, PLoS Global Public Health,

Please see the following as point-by-point responses to the comments given by the reviewers.

1. Title and Abstract
Line 7: under abstract : "compared between the first and later questionnaire": this appear vague, the authors should clarify the term "later questionnaire" specifically for easy reference.

Response: Thank you for this comment. We have revised the sentence as follows.

*“The changes in exercise habit, body mass index (BMI) and overweight status (BMI >25kg/m^2^) were compared between the first questionnaire and a questionnaire conducted a year later”* (Abstract line 7)

2. Data presented and analyzed in this study were post-covid , a brief background introductory information on the pre-covid lifestyle factors of exercise habits and overweight status among Japanese population would give readers a more robust understanding of the impact of this study. The authors may wish to consider this.

Response: We appreciate this important suggestion. We have referred the result of The National Health and Nutrition Survey in Japan 2019 and have added the following sentence in the result and limitation.

*“According to the National Health and Nutrition Survey in Japan 2019[24], about 33% of males and 29% of females had exercise habit of ≥ 2 days per week. Our data showed slightly higher percentage (36% in male and 34% in female) answered they had ≥ 2 days per week of exercise habit.”* (Page 13, Line 2-4 in the manuscript with track).

*“Indeed, our data showed the proportion of those who had exercise habit of ≥ days per week was higher than the that of The National Health and Nutrition Survey in Japan 2019, which may reflect these selection bias.* (Page29, Line 6-9 in the manuscript with track)

3. MATERIALS AND METHODS:
Line 7 : Online questionnaire were conducted five times.
Why five times? What was the rationale for this? Were the same questions administered five times on the same or different populations? How was the baseline data determined? Were there pre and post data comparison? Which of these represented the baseline/pre and post data collected? The authors may wish to clarify all these for easy comprehension.

Response: The times of the questionnaire was limited mainly due to financial reason, and we have added the following sentences.

“*The questionnaire was conducted only for a year mainly due to limited finance.”* (Page 5, Line 8 in the manuscript with track)

*“The number of questionnaires was also limited to five times from the same reason.”* (Page 28, Line 18-19 in the manuscript with track)

4. DEFINITION OF CHANGES IN THE EARLY AND LATE PHASES

Line 3: Reference was made to first, second and fifth questionnaire.
Was there a third and fourth questionnaire?
If the questionnaires were administered five times, an explanation or reference may be included in the study for third and fourth questionnaire.

Response: Thank you for this really important comment. We have added Table 2 that describes follow-up rate of the participants by sexes and age groups, and added the following sentences in the result and limitation.

*“Proportion of missing data in each questionnaire by sex and age category is shown in Table 2. The proportions increase with time and were higher among female participants and participants at younger age (≤30 years old).”* (Page13, Lin 9-11 in the manuscript with track)

“*Second, although the participants were matched to the national demographic background, dropout rate was different between sexes and age groups.*” (Page 28, Line 19 – Page 29, Line 1 in the manuscript with track)

5. TARGET POPULATION
Line 14 & 15: mentions selection of target population from the database, was there a scientific determination for this selection? How did you arrive at a sample size of 2000 for the study ( line 17). A simple clarification will be fine.

Response: As the participants were randomly selected among the registered monitors of NTTCom. We have added the following sentence.

“*The participants were Japanese people aged 18–74 years living in Japan who were randomly selected from the database of registered monitors of the NTTCom.*” (Page 5, Line 15-16 in the manuscript with track)

7. In addition to some risk factors mentioned, genetics is another major risk factor in being overweight.
The authors may wish to list out all the possible risk factors for being overweight but clarify that the focus of this study is on the impact of covid-19 pandemic.

Response: Thank you for these really useful comments. We have carefully looked at other literatures and have added the following sentences in discussion and limitation.

“*Previous　reports　suggest　that　prolonged　evacuation　may　increase　the　risk　of　chronic　conditions　including obesity, presumably due to increased mental　stress and poor access to healthcare services [25, 26]. Our research suggests that depression and anxiety had limited impact on the health problems, suggesting there might be other cause of health deterioration during the pandemic.*” (Page 23, Line 15-18 in the manuscript with track)

“*Finally, the survey did not include that of genetic factors, which may account for 40 to 50 % of variability in body weight status [41]. In addition, there might be difference in genetic backgrounds between Japan and other countries, which may limit the generalizability of our findings.*” (Page 29, Line 18 – Page 30, Line 2 in the manuscript with track)

Conclusion: The authors have addressed the concerns raised by the editor and previous reviewer. They may wish to address these fresh concerns I have raised. However, this research is massive and the authors have done a commendable work.

Response: According to the helpful advices from the reviwers, have added the following sentence in the last part of the conclusion.

“*Further research is still needed to clarify the detailed factors that affect exercise habits and overweight status, such as eating habits, change in the volume of housework, other causes of mental stress and genetic factors that may impact body weight status.*” (Page 30, Line 17-18 in the manuscript with track)
